# Possible Therapeutic Utility of anti-Cell Adhesion Molecule 1 Antibodies for Malignant Pleural Mesothelioma

**DOI:** 10.3389/fcell.2022.945007

**Published:** 2022-07-12

**Authors:** Man Hagiyama, Takahiro Mimae, Akihiro Wada, Fuka Takeuchi, Azusa Yoneshige, Takao Inoue, Naoyuki Kotoku, Hironobu Hamada, Yoshitaka Sekido, Morihito Okada, Akihiko Ito

**Affiliations:** ^1^ Department of Pathology, Kindai University Faculty of Medicine, Osaka, Japan; ^2^ Department of Surgical Oncology, Hiroshima University, Hiroshima, Japan; ^3^ Division of Molecular Pathology, Graduate School of Medical Science, Kindai University, Osaka, Japan; ^4^ College of Pharmaceutical Sciences, Ritsumeikan University, Shiga, Japan; ^5^ Department of Physical Analysis and Therapeutic Sciences, Graduate School of Biomedical and Health Sciences, Hiroshima University, Hiroshima, Japan; ^6^ Division of Cancer Biology, Aichi Cancer Center Research Institute, Nagoya, Japan

**Keywords:** cell adhesion molecule 1 (CADM1), neutralizing antibody, antibody drug complex, monomethyl auristatin E (MMAE), humanized antibody

## Abstract

Malignant pleural mesothelioma (MPM) is a highly aggressive malignant tumor, and the effective therapeutic drugs are limited. Thus, the establishment of novel therapeutic method is desired. Considerable proportion of MPMs are shown to express cell adhesion molecule 1 (CADM1), and to use CADM1 to bind to and proliferate on the pleural mesothelial surface, suggesting that CADM1 is a possible therapeutic target. Here, anti-CADM1 ectodomain chicken monoclonal antibodies, 3E1 and 9D2, were examined for their possible therapeutic utility. The full-length form of CADM1 was expressed in eight out of twelve human MPM cell lines. MPM cell lines were cultured on a confluent monolayer of mesothelial MeT-5A cells in the presence of 9D2, the neutralizing antibody. 9D2 suppressed the cell growth of CADM1-positive MPM cells with the loss and aggregation of CADM1 molecules on the MPM cell membrane, but not of CADM1-negative MPM cells. Co-addition of 3E1, lacking the neutralizing action, enhanced the growth-suppressive effect of 9D2. The two antibodies were tested as drug delivery vectors. 3E1 was converted into a humanized antibody (h3E1) and conjugated with monomethyl auristatin E (MMAE), a tubulin polymerization inhibitor. When the resulting h3E1–MMAE antibody-drug conjugate (ADC) was added to the standard cultures of CADM1-positive MPM cells, it suppressed the cell growth in a dose-dependent manner. Co-addition of 9D2 enhanced the growth-suppressive effect of h3E1–MMAE ADC. Anti-CADM1 ectodomain antibodies were suggested to serve as both antibody drugs and drug vectors in the treatment of MPM.

## Introduction

Malignant pleural mesotheliomas (MPMs) are diffuse tumors that primarily grow as a plaque and spread on the pleural surface (Tischoff et al., 2011), and generally bring a very poor prognosis. The available treatment options are surgical resection, chemotherapy, and radiation therapy in patients with MPM, and the multimodal treatment has been demonstrated to be the most effective strategy but benefits only selected patients. Most patients with MPM have extensive disease, that is, have no indication for surgical resection or radiation therapy, so that they receive palliative chemotherapy. The National Comprehensive Cancer Network guideline recommends pemetrexed, vinorelbine, and gemcitabine as second-line treatment options and pembrolizumab or nivolumab, alone or in combination with ipilimumab, as subsequent options, if not administered in the first line (Baas et al., 2021; Nowak et al., 2022). However, the effect of treatment for MPM beyond first-line therapy is still unsatisfactory. Thus, novel treatment strategy is desired.

Cell adhesion molecule 1 (CADM1) is an IgCAM-type intercellular adhesion molecule located on the lateral membrane of particular types of epithelial cells, including biliary duct and lung alveolar cells (Ito et al., 2003b and Ito et al., 2007), and is assumed to contribute to the integrity of epithelial cell morphology (Yageta et al., 2002; Sakurai-Yageta et al., 2009) and polarity (Shingai et al., 2003). When simple columnar epithelial cells proliferate and crowd, CADM1 accumulates on the lateral membrane, and thereby avoids the cells from apoptosis (Hagiyama et al., 2020). CADM1 is also expressed in non-epithelial cells, including nerves and mast cells (Biederer et al., 2002; Ito et al., 2003a; Hagiyama et al., 2009). Mast cells use CADM1 to adhere fibroblasts via its heterophilic *trans*-binding, and this adhesion helps mast cells proliferate or survive (Ito et al., 2003a and Ito et al., 2004). In the past, we generated two anti-CADM1 ectodomain chicken monoclonal antibodies, named 3E1 and 9D2 (Furuno et al., 2005; Koma et al., 2008). Both have different epitopes (Hagiyama et al., 2019). 9D2 has a neutralizing action, *i.e.*, it is able to block CADM1 *trans*-binding (Furuno et al., 2005).

Previously, we reported that considerable proportions of MPM expressed the full-length form of CADM1, and suggested that CADM1 should help MPM cells bind to and proliferate on the pleural surface composed of a mesothelial cell monolayer that lacks the full-length CADM1 (Ito et al., 2008). If 9D2 can neutralize CADM1 also in MPM cells, this antibody may suppress the growth of MPM cells on the pleural surface. 3E1 may play cooperative roles along with 9D2, because both can bind to the CADM1 ectodomain simultaneously (Hagiyama et al., 2019).

Here we aimed to disclose the potential for therapeutic modality of these anti-CADM1 antibodies on MPM. First, we confirmed that the majority of MPM cell lines expressed CADM1 and examined whether 9D2 would disturb CADM1 function in MPM cells. Next, we established a coculture that mimicked *in vivo* MPM cell plaque growth in the pleural cavity, and assessed the effects of 9D2 on the growth of MPM cells on a mesothelial cell monolayer. We also assessed the effects of 3E1 by co-addition. With bearing clinical use in mind, we finally converted 3E1 into a humanized antibody and conjugated it with monomethyl auristatin E (MMAE), a tubulin polymerization inhibitor (Bai et al., 1990) after the model of brentuximab vedotin (Adcetris; Francisco et al., 2003; Hamblett et al., 2004), and then examined whether the resulting antibody-drug conjugate (ADC) would suppress the growth of MPM cells, in combination with 9D2.

## Materials and Methods

### Cell Lines

The human MPM cell lines NCI-H28, NCI-H2052, NCI-H2452, MSTO-211H and MeT-5A cells were purchased from the American Type Culture Collection (Manassas, VA, United States), ACC-MESO-1 and ACC-MESO-4 cells were from Riken Cell Bank (Tsukuba, Japan). Y-MESO-8D, Y-MESO-9, Y-MESO-12, Y-MESO-14 cells were established by Sekido Y from patient tissue samples according to protocols approved by the Institutional Review Board, after written informed consent was obtained from each patient (Tanaka et al., 2017). These four lines were recently deposited at Riken Cell Bank (Tsukuba, Japan). An ACC-MESO-1 subline that expresses human CADM1 exogenously (MESO-1-CADM1) was established previously (Ito et al., 2008). The EHMES10 line was established by Hamada H (Nakataki et al., 2006). All cell lines were Mycoplasma-free, identified using short-tandem repeat analysis, and were cultured with Roswell Park Memorial Institute medium (RPMI-1640; Wako, Osaka, Japan) supplemented with 10% fetal bovine serum (FBS), antibiotics containing 100 unit/mL penicillin and 100 μg/ml streptomycin (Invitrogen, Carlsbad, CA, United States), and 5 mM HEPES buffer (Dojindo, Kumamoto, Japan) at 37°C in 5% CO_2_/95% air. All experimentation using these cell lines proceeded within 3 months or five passages after resuscitation.

### Antibodies

Antibodies against the CADM1 ectodomain (9D2 and 3E1, chicken monoclonal) were described previously (Koma et al., 2008), and were dissolved in phosphate-buffered saline (PBS) not containing azide. Other primary antibodies used were against CADM1 C-terminus (S4945; Sigma-Aldrich, St. Louis, MO), cell adhesion molecule 4 (CADM4) (MABN509; Sigma-Aldrich), and β-actin (Medical & Biological Laboratories, Nagoya, Japan). Antibodies against human IgG Fc and MMAE were purchased from Jackson ImmunoResearch (West Grove, PA) and Levena Biopharma (anti-vc-PAB-MMAE, LEV-PAE1; San Diego, CA, United States). Peroxidase- and fluorophore-conjugated secondary antibodies were obtained from Amersham (Bukinghamshine, England) and Jackson ImmunoResearch, respectively.

A control chicken IgY was purchased from R&D Systems (Minneapolis, MN, United States), and a normal human IgG from Fujifilm Wako Chemicals (Osaka, Japan).

### Western Blotting Analysis

Total 3 × 10^4^ cells of NCI-H28, NCI-H2052, NCI-H2452, MSTO-211H, MESO-1, MESO-1-CADM1, MESO-14, and EHMES10 cells; 8 × 10^4^ of MESO-4, MESO-8D, MESO-9 cells; 1 × 10^5^ of MESO-12 cells; and 6 × 10^4^ of MeT-5A cells were seeded onto a culture insert bottomed with a Matrigel-coated semipermeable membrane (Transwell, transparent PET membrane, pore size 0.4 μm; Falcon, Corning, Tokyo, Japan) placed in a 12-well plate. After 2 days, the cells cultured on a semipermeable membrane were reached 100% confluence and lysed in a buffer containing 50 mM Tris-HCl (pH 8.0), 150 mM NaCl, 1% Triton X-100 and 1 mM phenylmethylsulfonyl fluoride, and after removal of impurities by centrifugation, were subjected to Western blotting analyses as described in our previous report (Hagiyama et al., 2020; Yoneshige et al., 2021). Immunoreactive band intensities were quantified using ImageJ software (National Institutes of Health, Bethesda, MD, United States), as described previously (Mimae et al., 2012).

### Coculture of MPM Cells on MeT-5A Cell Monolayers

1.5 × 10^5^ of MeT-5A cells were seeded on Matrigel-coated coverslip-like-bottomed culture dishes of a 35-mm diameter (μ-Dish; ibidi, Munich, Germany). The next day, MeT-5A cells grew in a confluent monolayer, and the cell growth was halted by the treatment with mitomycin C (Fujifilm Wako Chemicals) at 10 μg/ml in CO_2_ incubator for one hour, followed by wash with culture medium three times. To label living cells with fluorescence, confluent MPM cells in standard culture dishes were incubated for 30 min in medium containing a fluorescent tracer DiI (Molecular Probe, Eugene, OR, United States) at a concentration of 10 mM, followed by wash with culture medium three times. Then, a scraper was used to peel off the cellular monolayer from the dish bottom gently and rigorously so as to break it into small sheets consisting of a few tens of cells. Cell sheets were harvested by gentle centrifugation, and seeded sparsely without overlapping onto MeT-5A cell monolayers treated with mitomycin C. To assess the growth kinetics of MPM cells, the cocultures were observed next day under a fluorescence microscope (CKX4; Olympus, Tokyo, Japan) equipped with a CCD camera (DP70; Olympus). Eight to 10 cell sheets labeled with DiI were selected randomly, and the images were captured to count the total number of cells making up individual sheets (Day 0). Then 9D2, 3E1, or 9D2 and 3E1 were added to the medium at a concentration of 1 μg/ml each. After 2 days of incubation, the cell sheets selected on Day 0 were observed again, and the images were captured to count the number of DiI-labeled cells (Day 2). For each sheet, a ratio of the cell number on Day 2 to that on Day 0 was calculated and expressed as the growth rate. The mean and standard deviation of the growth rate were calculated from eight to ten sheets for each experimental group. Control experiments were done using control IgY instead of 9D2 and 3E1 according to the same procedures. Experiments were repeated three times with similar results.

### Immunofluorescence and Confocal Microscopy

Immunofluorescence was performed as previously described (Ito et al., 2012; Mimae et al., 2014). Briefly, MPM cells were cocultured on MeT-5A cell monolayers for in the presence of antibodies for 2 days, as described above. The cocultures were fixed in 4% paraformaldehyde for 10 min at 4°C, blocked with 2% bovine serum albumin (BSA) for 30 min at room temperature, and incubated with 3E1 overnight at 4°C, then visualized with Alexa Flour 488-conjugated secondary antibody (anti-chicken IgY; Jackson ImmunoResearch). After washing with PBS three times, nuclei were labeled with DAPI (Molecular Probes) for 2 h at 4°C. Fluorescence images were captured using a C2+ confocal scanning system equipped with 488-nm argon and 543-nm helium-neon lasers (Nikon, Tokyo, Japan). The immunofluorescence experiments were repeated three times with similar results.

### Apoptosis Detection

DiI-labeled MPM cell sheets were cocultured on MeT-5A cell monolayers in the presence of either IgY or 9D2 and 3E1 (1 μg/ml each). After 2 days, apoptosis was detected by transferase-mediated dUTP nick-end labeling (TUNEL) method using the Click-iT™ TUNEL Alexa Fluor™ 488 Imaging Assay (ThermoFisher, Waltham, MA, United States) as described previously (Kato et al., 2018). Briefly, cells were washed with PBS, fixed in 4% paraformaldehyde for 15 min, and treated with 0.25% Triton X-100 in PBS. After washed twice with deionized water, the cells were incubated with the TUNEL reaction mixture containing terminal deoxynucleotidyl transferase 1 h at 37°C, followed by reaction with Alexa-488-labelled dUTP for 30 min at 37°C. Soon afterward, nuclei were counterstained with DAPI (Dojindo). Triple fluorescence images were captured using a C2+ confocal scanning system equipped with 488-nm argon and 543-nm helium–neon lasers (Nikon). A cell was deemed TUNEL-positive if it exhibited TUNEL signals among the DAPI nuclear stain. The number of TUNEL-positive cells was counted among 30–50 DiI-labeled MPM cells per MPM cell sheet. This measurement was performed in 10–15 MPM cell sheets in a μDish, and the mean and standard error of the proportion of TUNEL-positive cells were calculated for each experimental group. The TUNEL assays were repeated three times with similar results.

### Generation of Humanized Antibodies of 3E1

The procedures are described in Supplementary Methods. Nucleotide sequences are deposited in GenBank. Accession nos. for 3E1 are LC706201 and LC706202; and for a humanized antibody clone used here, LC706472 and LC706473.

### Generation of Antibody-Drug Conjugate

A precursor of ADC containing a maleimidocaproyl (mc) spacer, a cathepin-sensitive valine–citrulline dipeptide, a *p*-amino-benzyloxy carbonyl linker and a MMAE payload (mc-vc-PAB-MMAE) was purchased from MedChemExpress (Monmouth Junction, NJ, United States). 400 μL of PBS containing human IgG (hIgG) or human 3E1 (h3E1) at 0.5 mg/ml were put into a glass reaction vessel, and 8.2 μl of 1 mM EDTA aqueous solution and 1 μl of 2 mg/ml polysorbate Tween 20 aqueous solution were added to the antibody solution. Under a stirring condition, 2 μl of 10 mg/ml tris (2-carboxyethyl) phosphine hydrochloride aqueous solution was added at 24°C, then, the mixture was incubated at 37°C for 60 min to reduce inter-chain disulfide bonds of the antibody and cooled down on ice. Under stirring at 20°C, 12 μl of 1 mg/ml mc-vc-PAB-MMAE in 80% DMSO solution was added to the antibody solution three times, and then was incubated at the same temperature for 40 min to bind the drug linker precursor to the antibody. Next, 8.2 μl of 1 mg/mL N-acetylcysteine solution was added, and the mixture was stirred at 20°C for 30 min to stop the reaction of the drug linker precursor. The solution (∼500 μl) was put into an Amicon Ultra-50 kDa cutoff filter unit (Merck Millipore, Burlington, MA), and centrifuged to be reduced in volume by 40 μl. After adding 450 μl of PBS, the unit was centrifuged again likewise. This ultrafiltration and buffer replacement procedure was repeated ten times. The resulting final solution (∼40 μl) was diluted with ∼100 μl of PBS to contain the antibody conjugated with MMAE (hIgG–MMAE and h3E1–MMAE) at a concentration of 1 mg/ml. The drug antibody ratio (DAR) of the ADCs was accomplished by the standard ultraviolet-visible (UV-Vis) absorption spectroscopy (McDonagh et al., 2006). Briefly, the antibody and drug had distinct absorbance maxima (λ_max_ = 280 and 248 nm, respectively). Using absorbance measurements of ADCs and the extinction coefficients of the antibody and payload, the average DAR was determined.

### Water-Soluble Tetrazolium-8 Assay

Cell viability was assessed with the water-soluble tetrazolium-8 (WST-8)-based colorimetric assay using Cell Counting Kit 8 (Dojindo, Kumamoto, Japan) (Kimura et al., 2021; Hagiyama et al., 2022). MPM cells (1 × 10^3^ in 100 μl) were seeded in a 96-well plate in triplicate. Next day, when cells grew to 30% confluency, hIgG–MMAE ADC and control IgY, h3E1–MMAE ADC and control IgY, and h3E1–MMAE ADC and 9D2 were added to each well at indicated concentrations. After 5 days, cells were incubated with WST-8 for 40 min, and the absorbance at 450 nm was measured using an automated microplate reader. Measurement of mitochondrial dehydrogenase cleavage of WST-8 to formazan dye provided an indication of cell viability. To calculate half maximal inhibitory concentration (IC50) values, a 4-parameter logistic curve was drawn for each experimental group by using ImageJ software (https://imagej.nih.gov/ij/). Then, IC50 was calculated according the following equation: IC50 = 10^[Log (A/B) x (50 - C)/(D - C) + Log(B)], in which A, a higher concentration of two values that sandwich IC50; B, a lower concentration of two values that sandwich IC50; C, cell viability (%) at B; and D, cell viability (%) at A.

### Statistical Analysis

Cell growth rates and TUNEL assays were analyzed between two experimental groups using the paired two-tailed Student *t* test. Cell viability was analyzed using one-way ANOVA among all three groups, and the Bonferroni correction was applied to particular two groups. A *p*-value ≤ 0.05 was considered to indicate statistical significance.

## Results

### Cell Adhesion Molecule 1 Expression in MPM Cell Lines and Actions of 9D2 on It

**FIGURE 1:**
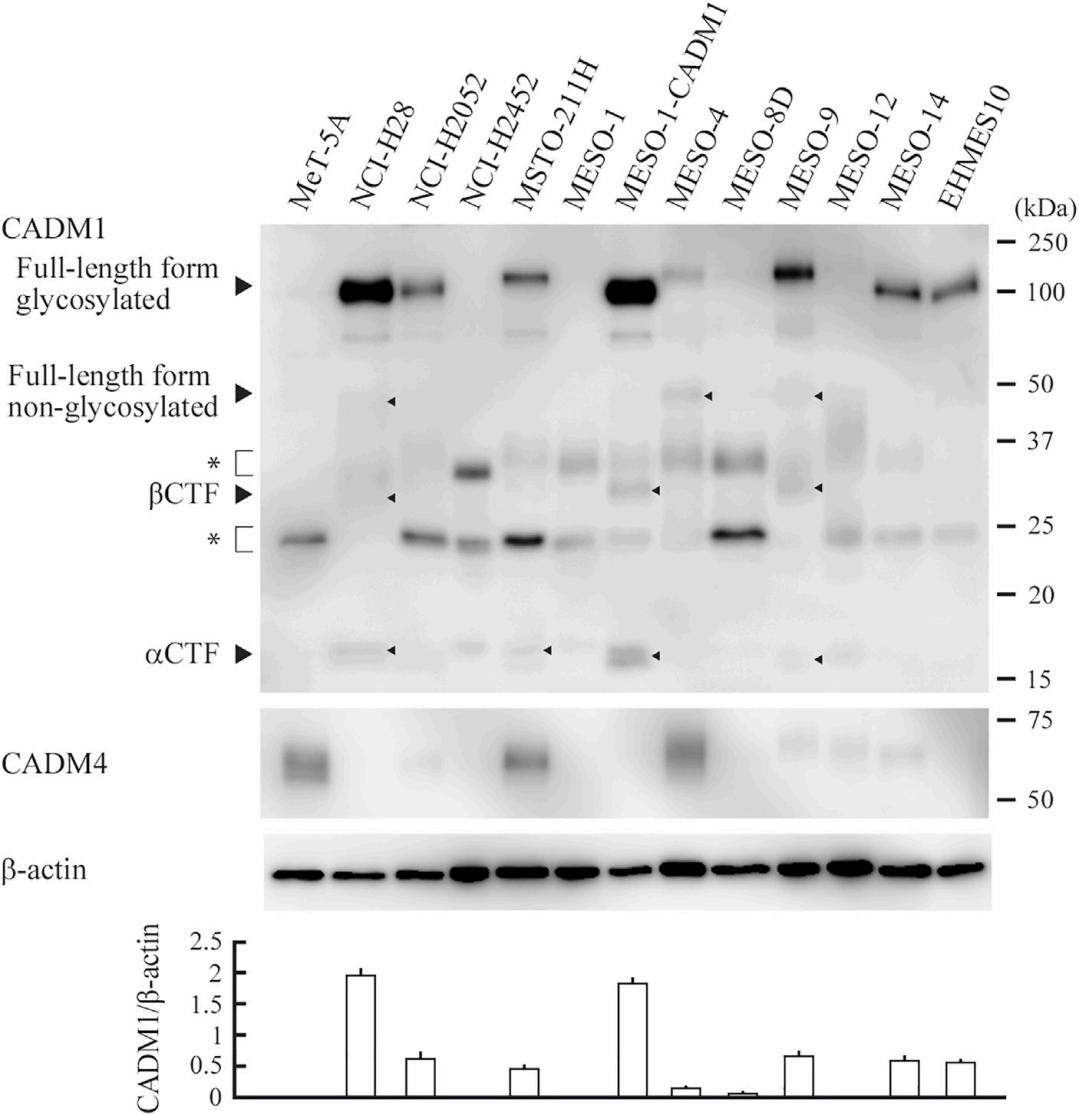
CADM1 protein expression in MPM and mesothelial cell lines. Cell lysates of twelve human MPM cell lines indicated and one mesothelial cell line MeT-5A were blotted with an anti-CADM1 C-terminal antibody (upper). Arrowheads depict various forms of CADM1; full-length forms glycosylated and non-glycosylated, C-terminal fragments generated by α- and β-ectodomain shedding (αCTF and βCTF). Asterisks indicate non-specific bands caused by the secondary antibody. After stripping, the blot was reprobed with antibodies against CADM4 (middle) and β-actin (lower) to indicate the expression of a heterophilic binding partner and the amount of protein loading per lane, respectively. Expression levels of the glycosylated full-length form relative to β-actin are plotted in a bar graph at the bottom. For each cell line, the mean value of three independent experiments is plotted with a thin line indicating the standard deviation, which is often too small to be shown by a line.

We performed Western blot analyses of eleven MPM cell lines, one subline with exogenous CADM1, MESO-1-CADM1, and one mesothelial cell line, MeT-5A. The full-length form of CADM1 was detected in 8 cell lines, with a large variation in the expression levels (NCI-H28, NCI-H2052, MSTO-211H, MESO-1-CADM1, MESO-4, MESO-9, MESO-14, and EHMES10; CADM1-positive; Figure 1). The C-terminal fragments generated by α- and β-ectodomain shedding, termed αCTF and βCTF (Nagara et al., 2012; Mimae et al., 2014), were also detected faintly in four CADM1-positive lines, NCI-H28, MSTO-211H, MESO-1-CADM1, MESO-9 (depicted by arrowheads in Figure 1). Normalized expression levels of the full-length CADM1 were shown in the bottom of Figure 1. MeT-5A mesothelial cells did not express the full-length CADM1, but expressed CADM4, a heterophilic *trans*-binding partner of CADM1 (Figure 1).

**FIGURE 2:**
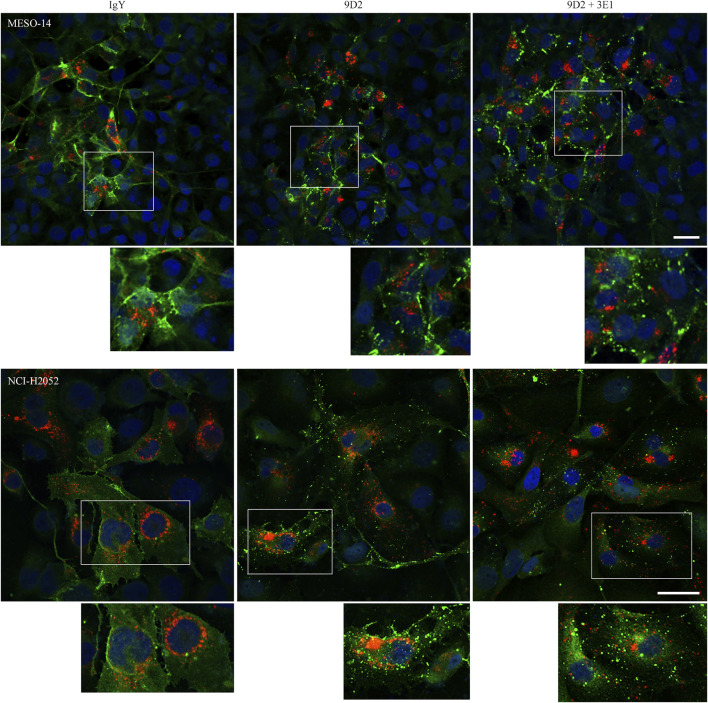
Immunofluorescence of CADM1 in MESO-14 and NCI-H2052 cells cultured on a MeT-5A cell monolayer in the presence of 9D2 and 3E1. MESO-14 and NCI-H2052 cell sheets were labeled with DiI (red) and were cocultured on a MeT-5A cell monolayer in the presence of control IgY (1.0 μg/ml; left), 9D2 (1.0 μg/ml; middle), or 9D2 and 3E1 (1.0 μg/ml each; right). After 2 days, the cocultures were immunostained with the anti-CADM1 antibody (green). Nuclei were counterstained with DAPI (blue). Images were captured by a confocal microscope; representatives are shown. Bar = 50 μm. In each photomicrograph, a boxed area is enlarged at the bottom for observation at single-cell level.

We established a coculture that mimicked an *in vivo* typical MPM growth pattern showing cellular plaque formation on the pleural surface. Small cell sheets of CADM1-positive MPM cells were labeled with cell membrane-impermeable fluorescent dye DiI in standard culture dishes, and transferred on a MeT-5A cell monolayer in Matrigel-coated μ-Dishes (Supplementary Figure S1). MESO-14 and NCI-H2052 cell sheets were subjected to this coculture, and were incubated in the presence of 9D2, 3E1, or control IgY. After 2 days, CADM1 localization was examined by immunofluorescence. In the coculture incubated with 3E1 or control IgY, CADM1 was detected clearly on the MPM cell membrane in a linear staining pattern and weakly in the cytoplasm in a diffuse pattern (Figure 2 and Supplementary Figure S2A). There was a clear difference in the coculture incubated with 9D2. CADM1 was detected in a coarse particle pattern on the cell membrane and in the cytoplasm, indicating aggregation of CADM1 proteins (Figure 2) Quite similar results were obtained in the coculture of two other CADM1-positive MPM cells (Supplementary Figure S2B). In the MESO-14 and NCI-H2052 cocultures, 9D2 and 3E1 was co-added to the medium. The coarse particle pattern for CADM1 immunostaining was detected more clearly (see enlarged views in Figure 2).

### Suppression of Malignant Pleural Mesothelioma Cell Growth by 9D2 and Cooperative Action of 3E1

**FIGURE 3:**
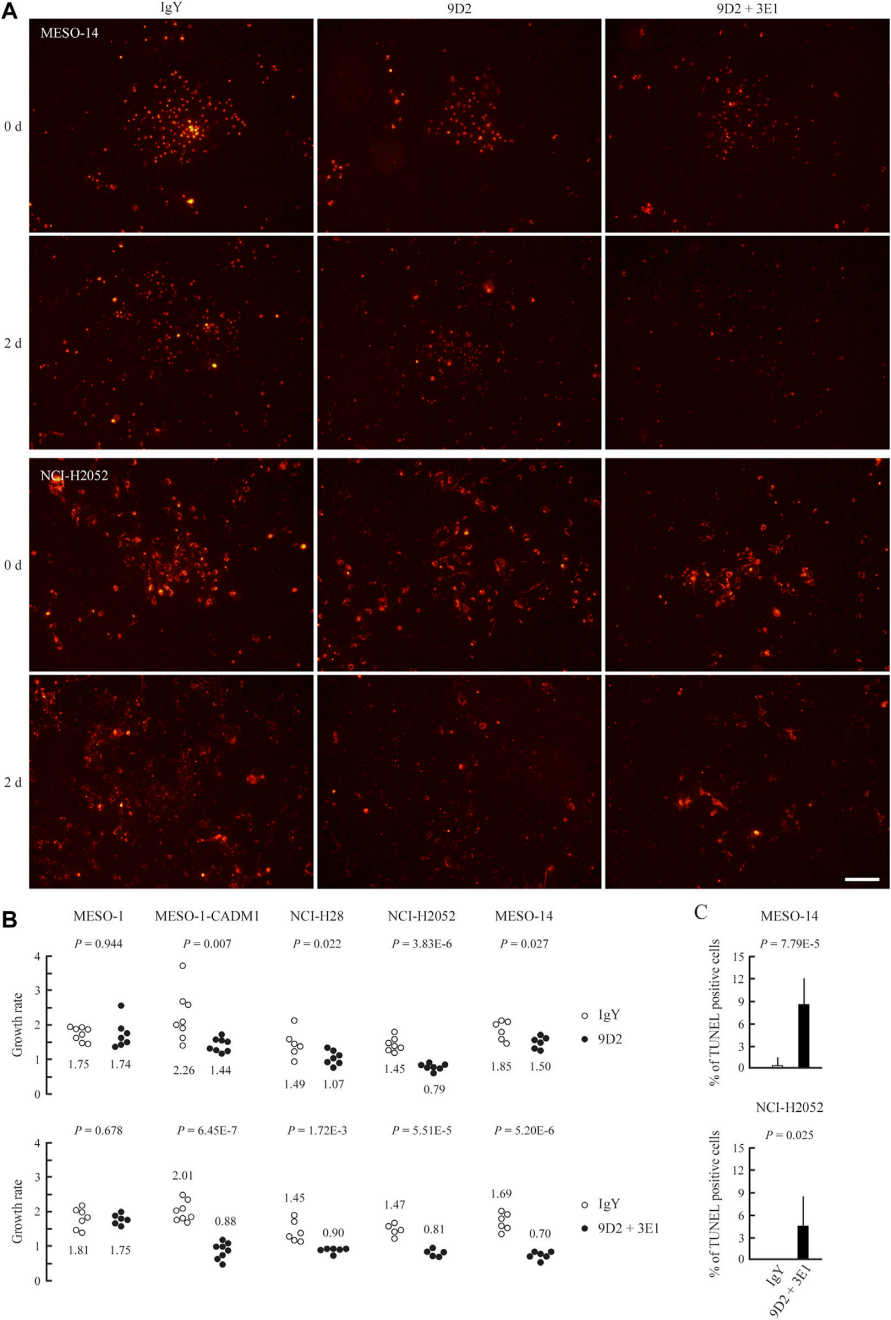
Growth suppression of MPM cells on MeT-5A cells by 9D2 and 3E1 **(A)** MESO-14 and NCI-H2052 cell sheets were labeled with DiI (red) and were cocultured on a MeT-5A cell monolayer in the presence of control IgY (1.0 μg/ml; left), 9D2 (1.0 μg/ml; middle), or 9D2 and 3E1 (1.0 μg/ml each; right). Cell sheets were observed through a fluorescence microscope at the time of antibody addition (0 d) and after 2 days (2 d) **(B)** MESO-1, MESO-1-CADM1, NCI-H28, NCI-H2052, and MESO-14 cell lines were cocultured on a MeT-5A cell monolayer in the presence of either control IgY (1.0 μg/ml) or 9D2 (1.0 μg/ml) (upper graph), and in the presence of either control IgY (2.0 μg/ml) or 9D2 and 3E1 (1.0 μg/ml each) (lower graph). For individual cell sheets, the ratio of the cell number at the time of antibody addition (0 d) to that after 2 days (2 d) was calculated, and plotted as the growth rate in a dot graph. The mean was calculated from the ratios of five to ten sheets for each coculture group, and is shown below or above the dots. *p*-values are shown at the top of each graph. Bar = 100 μm **(C)** TUNEL assays of MESO-14 and NCI-H2052 cells cocultured on a MeT-5A cell monolayer for 2 days in the presence of either control IgY or 9D2 and 3E1. The mean proportion of TUNEL-positive MPM cells (%) is plotted with a thin line indicating the standard deviation. A *p*-value between two groups is shown at the top of each graph.

We assessed the growth rate of MPM cells in the coculture. We calculated the ratio of MPM cell numbers at the time of antibody addition and after 2 days for individual cell sheets. MPM cells were identified by pre-labeling with DiI. When cocultured with control IgY or 3E1, MPM cells grew for 2 days at a rate of 1.47–2.26 (Figure 3, and Supplementary Figure S3). In contrast, incubation with 9D2 significantly reduced the growth rate of CADM1-positive MPM cells, *i.e.*, MESO-1-CADM1, NCI-H28, NCI-H2052, and MESO-14, but not that of MESO-1 (Figure 3).

We co-added 9D2 and 3E1 to the culture medium. The growth rate fell below 1.0 in all four CADM1-positive cell lines, ranging from 0.70 to 0.90, whereas the co-addition did not impact on the growth rate of MESO-1 (Figure 3).

Cocultured MESO-14 and NCI-H2052 cells were subjected to TUNEL assays. Substantially no MPM cells were TUNEL-positive in the presence of control IgY, whereas the co-presence of 9D2 and 3E1 caused MPM cells to be TUNEL-positive at a rate of approximately 4–8% (Figure 3C and Supplementary Figure S4).

### 3E1-Based Antibody-Drug Conjugate Effectively Reduces Malignant Pleural Mesothelioma Cell Viability

**FIGURE 4:**
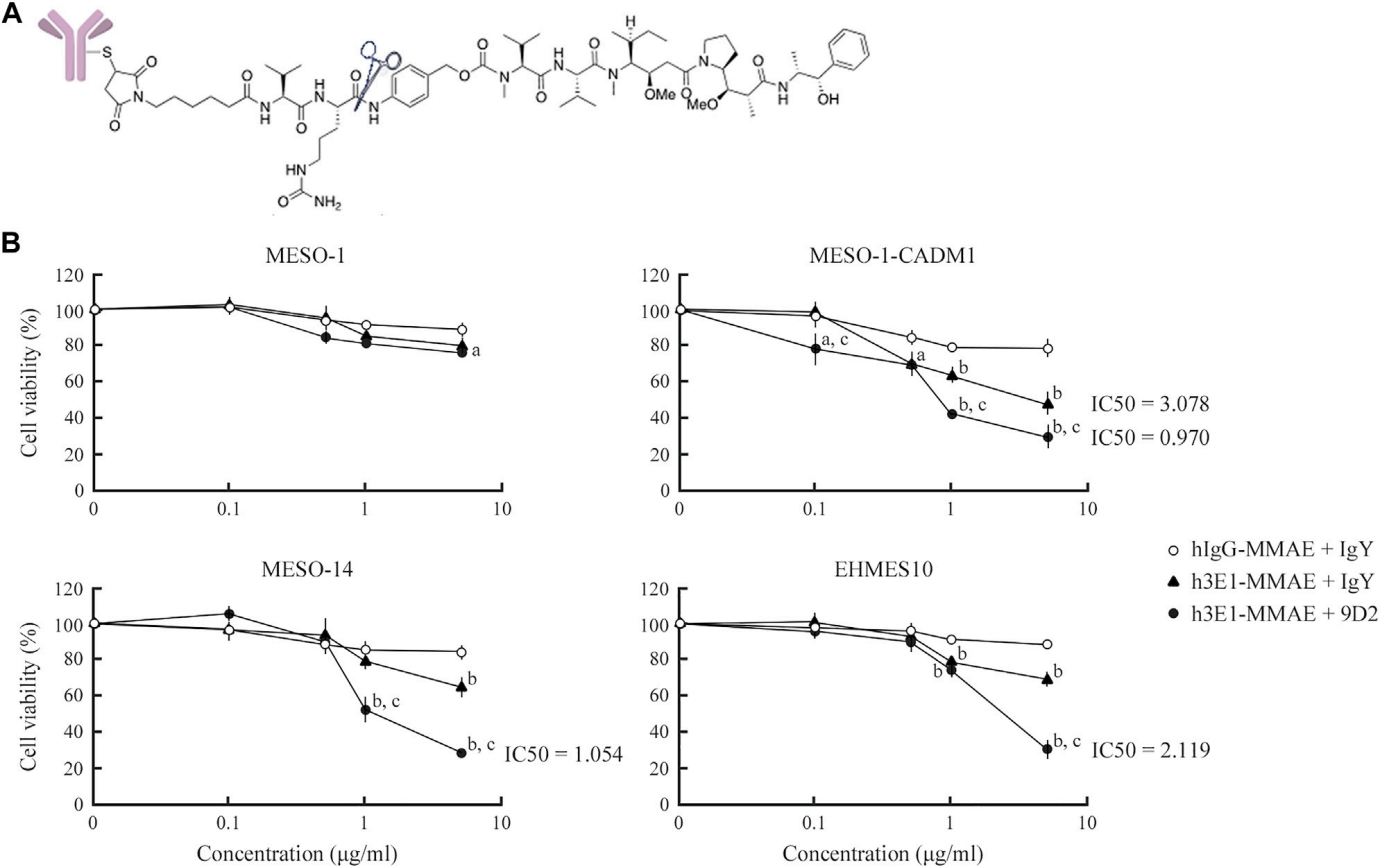
Killing of MPM cells by h3E1–MMAE ADC **(A)** Structure of h3E1–MMAE ADC. The cathepsin cleavage site is indicated by scissors **(B)** MPM cells were cultured in a 96-well plate at 30% confluence, and either hIgG–MMAE or h3E1–MMAE ADC was added along with wither control IgY or 9D2 at indicated concentrations. After 5 days, cell viability was calculated by WST-8 assays in triplicate. The mean is plotted in a line graph, with a thin vertical line indicating the standard deviation. ^a^ and ^b^, *p*-value < 0.05 and 0.01, respectively, when compared with the value of hIgG–MMAE ADC + IgY at the identical concentration. ^c^, *p*-value < 0.01, when compared with the value of h3E1–MMAE ADC + IgY at the identical concentration.

Through generation and screening of a single chain variable fragment library for 3E1, we obtained several clones of the humanized 3E1 antibody in an IgG backbone (Supplementary Methods). We screened the clones by ELISA using the recombinant CADM1 ectodomain, and found a clone, called h3E1 here, that showed a binding affinity as high as 3E1 (Supplementary Figure S3). We conjugated h3E1 with MMAE, an anti-cancer drug, via a linker containing the peptide-mimic sequence cleavable by cathepsin (Figure 4 and Supplementary Figure S4). The resulting ADC is structurally same as brentuximab vedotin, in which an anti-CD30 antibody, not h3E1, is used (Francisco et al., 2003; Hamblett et al., 2004). For control, human IgG was conjugated with MMAE according to the same procedures. We subjected the ADCs to Western blot analyses using an anti-MMAE antibody, and found that the immunoreactive bands were searing and were shifted to higher molecular weight forms, suggesting successful conjugation of the antibodies with ADC precursor (Supplementary Figure S4). UV-Vis spectroscopy revealed that DAR was 2.86 for hIgG–MMAE, and 2.57 for h3E1–MMAE.

Four MPM cell lines, MESO-1, MESO-1-CADM1, MESO-14, and EHMES10, were seeded in a 96-well plate, and were cultured in the presence of h3E1–MMAE ADCs and either control IgY or 9D2 at various concentrations, *i.e.*, 0.1, 0.5, 1, and 5 μg/ml each. For control, cells were cultured in the presence of hIgG–MMAE and control IgY. After 5 days, the cell viability was assessed by WST-8 assays. In the absence of 9D2, h3E1–MMAE ADC reduced the cell viability of CADM1-positive MPM cells by approximately 30–50% in a dose-dependent manner, with IC50 = 3.078 μg/ml for MESO-1-CADM1 (Figure 4). Co-presence of 9D2 further decreased the viability below 30%, and resulted in IC50 = 0.970 and 1.054 μg/ml for MESO-1-CADM1 and MESO-14, respectively (Figure 4). h3E1–MMAE ADC was substantially ineffective on MESO-1 (Figure 4).

## Discussion

The expression of the full-length form of CADM1 was detected in more than half of MPM cell lines examined here. This is consistent with our past clinical study reporting that a quarter of MPM cases expressed the full-length CADM1 abundantly, and some of the rest cases expressed it at low but detectable levels (Ito et al., 2008). In the coculture mimicking *in vivo* growth of MPM plaques on the pleural surface, MESO-1-CADM1 cells grew more than the original MESO-1 cells, suggesting that CADM1-mediated cell adhesion was involved in MPM cell growth on mesothelial cells. Actually, 9D2 suppressed the growth of CADM1-positive MPM cells in association with aggregation of CADM1 proteins. Heterophilic *trans*-binding between CADM1 and CADM4 was assumed to help CADM1-positive MPM cells adhere to and grow on a MeT-5A cell monolayer. Notably, 9D2 showed a larger growth-suppressive effect on NCI-H2025 than on NCI-H28, despite the fact that the 2 cell lines expressed CADM1 at low and high levels, respectively. This suggests that CADM1, even though expressed weakly, may be the important adhesive machinery for MPM to grow on the pleural surface. Especially, when co-added with 3E1, 9D2 decreased the growth rates below one in all four CADM1-positive MPM cells (Figure 3B), probably inducing MPM cell death. One of the mechanisms appeared to be apoptosis (Figure 3C and Supplemtary Figure S4). Therefore, 9D2 could be a promising anti-MPM drug, which is supposed to be safely injected into the intrathoracic cavity for patients with CADM1-positive MPM, because the pleura is negative for the full-length form of CADM1 (Ito et al., 2008).

Though 3E1 alone did not affect CADM1 subcellular localization or MPM cell growth, it had a higher affinity to CADM1 than 9D2 (Supplementary Figure S5). Since this property may assure 3E1 of its efficient internalization into cells (Opaliński et al., 2018), we humanized 3E1 first, and generated h3E1–MMAE ADC after the model of brentuximab vedotin. While proceeding with the present study, we realized that our ADC is quite similar to enfortumab vedotin (Challita-Eid et al., 2016), because both ADCs are based on antibodies against intercellular adhesion molecules, CADM1 and Nectin-4, respectively, and these two adhesion molecules structurally resemble each other, especially in the ectodomain consisting of three Ig-like loops (Fabre et al., 2002; Mandai et al., 2015). Enfortumab vedotin is a recently developed ADC that mainly targets urothelial carcinoma (Challita-Eid et al., 2016; Wong and Rosenberg, 2021). Evidence is rapidly accumulating that this ADC is very effective in clinical practice (Rosenberg et al., 2019; Powles et al., 2021; Yu et al., 2021). This may be because Nectin-4 is easily internalized into the cell in response to the antibody binding, and ADC is efficiently internalized along with Nectin-4. Actually, when 9D2 was added to the MPM–MeT-5A coculture, CADM1 proteins appeared to be aggregated and localized not only on the cell membrane but also in the cytoplasm (Figure 2). In general terms, adhesion molecule members may be good for cell surface targets in the field of ADC development.

3E1 enhanced the growth-suppressive effect of 9D2, and 9D2 enhanced the cell killing activity of h3E1–MMAE ADC. Simultaneous binding of two antibodies to the CADM1 ectodomain may promote CADM1 aggregation and CADM1 internalization. Actually, more CADM1 aggregation particles were detected in the cytoplasm when 9D2 and 3E1 were co-added to the culture medium (Figure 2). Considering that 9D2 is a neutralizing antibody, this fact may allow us to speculate that 9D2 binding to CADM1 results in unbinding of CADM1 *trans*-binding between neighboring cells, followed by aggregation of CADM1 proteins, and internalization of CADM1–3E1 complexes. According to this scenario, neutralizing antibodies may serve as an enhancer of ADC efficacy in general.

Several kinds of ADCs have been developed for treatment of MPM. Anetumab ravtansine (BAY 94–9343) is one of the most studied, and is comprised of an anti-mesothelin antibody conjugated to MMAE (Golfier et al., 2014; Lambert and Morris, 2017). The *in vitro* IC50 is 5.72 nM for NCI-H226, a MPM cell line expressing mesothein (Golfier et al., 2014). Other ADCs are targeting epidermal growth factor receptor and trophoblast glycoprotein 5T4, and also carry MMAE (Schunselaar et al., 2018; Chia et al., 2020). IC50s of these ADCs are ∼8 μg/ml or more for MPM cell lines expressing the corresponding targets sufficiently (Schunselaar et al., 2018; Chia et al., 2020). IC50 of h3E1–MMAE ADC (MW ∼ 153,000) was ∼1 μg/ml (∼6.5 nM) for MESO-1-CADM1 and MESO-14 and ∼2 μg/ml for EHMES10 in the presence of 9D2, and was ∼3 μg/ml for MESO-1-CADM1 in the absence of 9D2. These values are comparable with or even smaller than those reported so far. Noteworthy, the expression levels of CADM1 were moderate or low in MESO-14 and EHMES10. Co-administration of 9D2 might be a promising option, when CADM1-low MPM patients would be treated with h3E1–MMAE ADC.

At present, we do not know biological or clinical differences between CADM1-positive and -negative MPMs, but the present study suggests that CADM1 is an Achilles’ heel of MPM if the MPM expresses the full-length CADM1. We are now generating humanized antibodies of 9D2, and planning animal experiments with orthotopic implantation of MPM cells and injection of h3E1–MMAE ADC and a humanized 9D2 antibody.

In conclusion, the present study not only highlights an essential role of cell adhesion in MPM growth, but also demonstrates that the anti-CADM1 ectodomain antibodies can serve as a new modality for MPM treatment. The antibodies appeared to be potentially useful as both antibody drugs and drug vectors. Further characterization of the antibodies may lead to development of a new therapeutic strategy for MPM.

## Data Availability

The datasets presented in this study can be found in online repositories. The names of the repository/repositories and accession number(s) can be found below: Protein Data Bank Japan, accession numbers: LC706472, LC706473, LC706201, LC706202
