# Supplementary material for: Possible Therapeutic Utility of anti-Cell Adhesion Molecule 1 Antibodies for Malignant Pleural Mesothelioma
Source: Front Cell Dev Biol. 2022 Jul 12;10:945007. doi: 10.3389/fcell.2022.945007 (PMC9315061; doi:10.3389/fcell.2022.945007)
Supplement: Supplementary file 1 [file DataSheet1.DOCX]

Revised manuscript 945007

**Supplementary material**

**Supplementary Methods**

**Humanized antibodies of 3E1**

**Primary single chain variable fragment (scFv) antibody library**

DNA fragments for variable region domains of the heavy and light chains were designed as follows, and were synthesized and PCR-amplified.

1. Segments for CDR1, CDR2, and CDR3 of the 3E1 cDNA (accession no. LC706201, and LC706202) were introduced into the framework sequence that was deduced from a human immunoglobulin germline (IGHV3-23).

2. In the Vernier zone codons that encode different amino acid residues between 3E1 and human IgG, the codons were modified to express both residues.

The resulting double stranded DNAs were inserted into pPDS (hCκ) phagemid vector (Yamanaka et al., 1995) via the EagI and BssHII sites. Escherichia *coli* XL1-Blue (200228, Stratagene, Agilent, Santa Clara, CA, USA) was transformed by electroporation with the phagemid vectors, and cultured in SOC medium supplemented with 2% glucose for 5 hours at 37℃. After 1 hour of incubation in the presence of helper phages, bacteria were collected by centrifugation. The bacteria were resuspended in 2×YT medium containing 100 μg/mL IPTG, and incubated overnight at 37℃. Next day, after filtration, a 20% PEG/2.5 M NaCl solution was added to the supernatant that contained phages expressing scFv antibodies at a ratio of 1:5 in volume. After being left on ice for 1 hour, the supernatant was centrifuged for 20 min at 10,000×*g*. The precipitates were resuspended in phosphate-buffered saline (PBS) and stored at 4℃ (primary scFv antibody library).

**Panning and clone selection**

Specific phagemid clones were selected by panning as follows. Immunoplates (MaxiSorp, Nunc, ThermoFisher Scientific, Tokyo, Japan) were coated with the CADM1 ectodomain recombinant protein (1 μg/mL) (Koma et al., 2004) overnight at 4℃, and next day, were blocked with 25% blocking solution (UKB80; KAC Co., Ltd., Kyoto, Japan) for 1 hour at 37℃. Forty μL of the scFv library and 360 μL of 25% blocking solution (UKB80; KAC Co., Ltd., Kyoto, Japan) were mixed, and after left for 30 min, were transferred into each Immunoplate well, then left for 1 hour at 37℃. After wash with PBS-T 5 times, log phase XL1-Blue was added to each well and incubated for 1 hour at 37℃. Bacteria were removed, and 50 μL of Gly-HCl (pH 2.2) was added to each well. After 5 min, the well solution was neutralized with Tris-HCl (pH 9.0), and collected. The solution was mixed with the XL1-Blue removed in the previous procedure, and were cultured for 30 min at 37℃ with shaking at 200 rpm. Then, glucose (final concentration 2%) was added to the medium, and the culture was continued for 1 hour and for another 1 hour in the presence of helper phases. Bacteria were collected by centrifugation, and resuspended in 2×YT medium containing 100 μg/mL of IPTG, followed by overnight incubation at 37℃. Next day, the supernatant was collected, and after filtration, a 20% PEG/2.5 M NaCl solution was added to the supernatant that contained phages expressing single chain variable fragment (scFv) antibodies at a ratio of 1:5 in volume. After being left on ice for 1 hour, the supernatant was centrifuged for 20 min at 10,000×*g*. The precipitates were resuspended in PBS and stored at 4℃ (panned scFv antibody library). The panning process was repeated five times.

The panned scFv library was mixed with log phase XL1-Blue, and seeded in a 2×YT plate for individual clones to make colonies separately. Colonized clones were cultured in 2×YT medium overnight at 37℃, and scFv antibodies were expressed by incubation overnight at 30℃ in the presence of helper phages with IPTG. The medium was centrifuged for 10 min at 3,500 rpm, and the supernatants were examined for their ability to bind to the immunoplates (MaxiSorp, Nunc) coated with the CADM1 ectodomain recombinant protein and blocked with 25% blocking solution (UKB80; KAC Co., Ltd., Kyoto, Japan), by ELISA. The supernatants were incubated in the plates for 1 hour at 37℃, and after wash with PBS supplemented with 0.1% Tween (PBS-T), were reacted with HRP-conjugated anti-human κ light chain antibody (1,000-fold dilution; ThermoFisher Scientific) for 1 hour at 37℃. After wash with PBS-T, coloring reaction was achieved with TMB (52-00-02; Kirkegaard & Perry Laboratories, Inc., Milford, MA, USA) for 10 min. Then, TMB stop solution (50-85-05; Kirkegaard & Perry Laboratories, Inc.) was added, and the coloring was detected and measured with a plate reader (BioTek Cytation 5; Agilent). Clones were selected that had high affinity to CADM1 but no affinity to BSA.

**Generation and purification of bivalent antibody**

cDNA fragments for variable region domains of the heavy and light chains were obtained by PCR using the plasmid from selected clones, and were inserted into the pcDNA3.4 expression vector (Invitrogen, ThermoFisher Scientific) that had already carried the cDNA for human IgG4 constant region of either heavy or light κ chain, using Seamless Cloning and Assembly Enzyme Mix (A14606; ThermoFisher Scientific). The heavy chain construct had one amino acid substitution from Ser to Pro in the core hinge region so as to be more stable as a bivalent antibody (Angalet, Mol Immunol 1993). Sequencing confirmed there were no mutations. The plasmid vectors were amplified and purified according to the standard methods, and were transiently expressed using Expi293 Expression System (A14635; ThermoFisher Scientific). After cultivation, the supernatant was collected by centrifugation for 5 min at 1,200 rpm, and was subjected to another centrifugation for 5 min at 3,000 rpm, then filtered. Antibody purification was done using MabSelect SuRe (17543802; Cytiva, Marlborough, MA, USA) according to the manufacturer’s instructions. The buffer was replaced with Dulbecco PBS using PD-10 columns (Cytiva), and the antibody concentration was adjusted to 3.0 mg/mL.

Purified antibodies were examined for their ability to bind to the immunoplates (MaxiSorp, Nunc) coated with the CADM1 ectodomain recombinant protein and blocked with 25% blocking solution (UKB80; KAC Co., Ltd.), by ELISA (Fig. S3). Antibody solutions were incubated in the plates for 1 hour at 37℃, and after wash with PBS-T, were reacted with HRP-conjugated anti-human κ light chain antibody (1,000-fold dilution; ThermoFisher Scientific) for 1 hour at 37℃. After wash with PBS-T, coloring reaction was achieved with TMB (52-00-02, Kirkegaard & Perry Laboratories, Inc., Milford, MA, USA) for 10 min. Then, TMB stop solution (50-85-05; Kirkegaard & Perry Laboratories, Inc.) was added, and the coloring was detected and measured with a plate reader (BioTek Cytation 5; Agilent). One of the antibodies with high affinity to CADM1 was used in the present study (accession no. LC706472, and LC706473). For reference, 3E1 and 9D2 were subjected to ELISA according to the procedures same as the above-mentioned, except that HRP-conjugated anti-chicken IgG (H+L) antibody (1,000-fold dilution; 5220-0373, Kirkegaard & Perry Laboratories) was used for detection instead of HRP-conjugated anti-human κ light chain antibody.

**Supplementary Figure Legends**

**Supplementary Figure S1.** Coculture of MPM cell sheets on a MeT-5A cell monolayer.

(A) Schematic presentation of the coculture.

(B) Representative images of the coculture.

MPM cells were pre-labeled with DiI, and the small cell sheets were seeded onto a MeT-5A cell monolayer treated with mitomycin C. After 4 hours, differential interference contrast (DIC; left) and fluorescent (right) images were captured. MPM cell sheets are circled. Bar = 100 μm.

**Supplementary Figure S2.** Immunofluorescence of CADM1 in the coculture of MPM cell sheets on a MeT-5A cell monolayer.

(A) NCI-H28 and MSTO-211H cell sheets were labeled with DiI (red) and were cocultured on a MeT-5A cell monolayer in the presence of either control IgY (left) or 9D2 (right) at a concentration of 1 μg/mL. (B) MESO-14 and NCI-H2052 cell sheets were labeled with DiI (red) and were cocultured on a MeT-5A cell monolayer in the presence of either control IgY (left) or 3E1 (right) at a concentration of 1 μg/mL. After 2 days, the cocultures were immunostained with the anti-CADM1 antibody (green). Nuclei were counterstained with DAPI (blue). Images were captured by a confocal microscope; representatives are shown. Bar = 50 μm.

**Supplementary Figure S3.** No growth suppression of MPM cells on MeT-5A cells by 3E1 alone.

(A) MESO-14 cell sheets were labeled with DiI (red) and were cocultured on a MeT-5A cell monolayer in the presence of either control IgY (1.0 μg/mL; left) or 3E1 (1.0 μg/mL; right). Cell sheets were observed through a fluorescence microscope at the time of antibody addition (0 d) and after 2 days (2 d). Bar = 100 μm. (B) For individual cell sheets, the ratio of the cell number at the time of antibody addition (0 d) to that after 2 days (2 d) was calculated, and plotted as the growth rate in a dot graph. The mean was calculated from the ratios of five to ten sheets for each coculture group, and is shown below the dots. A P-value is shown at the top of the graph.

**Supplementary Figure S4.** TUNEL assays of MESO-14 (upper) and NCI-H2052 (lower) cells cocultured on a MeT-5A cell monolayer for 2 days in the presence of either control IgY (left) or 9D2 and 3E1 (right).

MPM cells are labeled with DiI (red). Nuclei are counterstained with DAPI (blue). TUNEL signals are detected with Alexa Fluor™ 488 (green). Triple fluorescence images were captured by a confocal microscope; representatives are shown. Arrows depict TUNEL-positive MPM cells. Bar = 50 μm.

**Supplementary Figure S5.** ELISA of 3E1, the humanized clone h3E1 (upper), and 9D2 (lower).

The procedures are described in Supplementary Methods in detail. The antigens used in the left and right graphs are the recombinant CADM1 ectodomain (Koma et al., 2004) and bovine serum albumin (BSA), respectively. Note that the detection antibodies for 3E1 and h3E1 are different, *i.e.*, anti-chicken and anti-human, respectively. For 9D2, the detection antibody is the same as that for 3E1.

**Supplementary Figure S6.** Generation of h3E1–MMAE ADC.

(A) Structure of h3E1–MMAE ADC and release of MMAE from ADC.

(B) Western blot analyses of ADCs.

Fifty ng of human IgG (hIgG), h3E1, and their ADCs were electrophoresed on SDS-PAGE gels, and were plotted with antibodies against hIgG Fc fragment (left) and MMAE (right).

**References**

Koma, Y., Ito, A., Wakayama, T., Watabe, K., Okada, M., Tsubota, N., et al. (2004). Cloning of a soluble isoform of the SgIGSF adhesion molecule that binds the extracellular domain of the membrane-bound isoform. *Oncogene* 23(33)**,** 5687-5692. doi: 10.1038/sj.onc.1207761.

Yamanaka, H.I., Kirii, Y., and Ohmoto, H. (1995). An improved phage display antibody cloning system using newly designed PCR primers optimized for Pfu DNA polymerase. *J Biochem* 117(6)**,** 1218-1227. doi: 10.1093/oxfordjournals.jbchem.a124847.
